# Thin‐layer drying of parchment *Arabica coffee* by controlling temperature and relative humidity

**DOI:** 10.1002/fsn3.1144

**Published:** 2019-07-31

**Authors:** Sutida Phitakwinai, Sirichai Thepa, Wanich Nilnont

**Affiliations:** ^1^ Division of Energy Technology, School of Energy, Environment and Materials King Mongkut’s University of Technology Thonburi Bangkok Thailand; ^2^ Department of Mechanical Engineering, Faculty of Engineering and Architecture Rajamangala University of Technology Suvarnabhumi Nonthaburi Thailand

**Keywords:** drying model, effective moisture diffusivity, parchment coffee, thin‐layer drying

## Abstract

This paper presents thin‐layer drying of parchment coffee (*Coffea arabica*). Thin‐layer drying of parchment coffee was conducted under controlled temperatures (50°C, 60°C, and 70°C) and relative humidities (10%–30%). The temperature of the drying air was important for drying at a high temperature, which results in the rapid removal of moisture and reduced time for drying. Nine thin‐layer drying models (Newton, Page, Henderson and Pabis, logarithmic, two‐term, modified Henderson and Pabis, two‐term exponential, approximation diffusion, and modified‐Midilli) were fitted to the experimental data for parchment coffee. The drying parameters of parchment coffee were related to temperature and relative humidity. The best model was the modified‐Midilli model, which can be used to design the optimal dryer. The effective moisture diffusivity of parchment coffee drying was determined by minimizing the sum of squares of the deviations between the experimental data for the moisture content and the predicted values of thin‐layer drying. The effective moisture diffusivity as a function of the temperature at each relative humidity was expressed by the Arrhenius‐type equation.

## 1 INTRODUCTION

Coffee consumption is likely to increase, so the demand for coffee beans, which are the main raw materials of coffee, is increasing (International coffee organization, 2016, 2018). Coffee is also a valuable commercial crop in many countries of Southeast Asia (Vietnam, Indonesia, Malaysia, Laos, and Thailand). Thailand is ranked 4th of the Southeast Asian countries that export coffee (United States Department of Agriculture, 2018). The postharvest process of coffee cherries that become green coffee beans has several processes. Before the green coffee beans are sold and shipped, coffee cherries are peeled, the mucilage is removed, and then, the beans are considered parchment coffee. Then, parchment coffee, which consists of parchment and bean, will be dried by various methods. The drying process is an important part of food preservation. The purpose of drying is to use the osmotic process so that water evaporates from the product because this kind of water is useful for fungi and bacteria. In addition, drying makes the product lighter, resulting in a longer shelf life, and less space needed for storage and increased convenience in terms of transportation. To best understand the transfer processes during drying, it is essential to understand thin‐layer drying characteristics.

Thin‐layer drying is widely used to determine the drying kinetics of crops and has been carried out in various types of dryers (Alara, Abdurahman, Mudalip, & Olalere, 2018; Jiang et al., 2017; Lee & Kim, 2008). Many researchers have studied and predicted the drying behavior of a product to design a dryer using thin‐layer drying mathematical modeling (Aidani, Hadedkhodaparast, & Kashaninejad, 2016; Akpinar, Midilli, & Bicer, 2003; Asiru, Raji, Igbeka, & Elemo, 2013; Janjai, Intawee, Kaewkiew, Sritus, & Khamvongsa, 2011; Mabrouk, Benali, & Oueslati, 2012; Mahjoorian et al., 2017; Mujaffar & John, 2018; Naderinezhad, Etesami, Najafabady, & Falavarjani, 2016; Younis, Abdelkarim, & El‐Abdein, 2018).

Many studies have investigated the coffee drying process using several drying methods, such as solar drying (Deeto, Thepa, Monyakul, & Songprakorp, 2018), convective drying (Burmester & Eggers, 2010; Muhidong Mursalim, & Rahman, 2013; Nilnont et al., 2012; Siqueire et al., 2017), and hot air‐assisted microwave drying (Ghosh, & Venkatachalapathy, 2014). However, the purposes of this study are as follows: (a) to study the effect of temperature and relative humidity on the drying characteristics of parchment coffee, (b) to develop an appropriate thin‐layer drying model of parchment coffees, and (c) to evaluate the effective moisture diffusivity of parchment coffee.

## 2 MATERIALS AND METHODS

### 2.1 Experimental study

**Figure 1:**
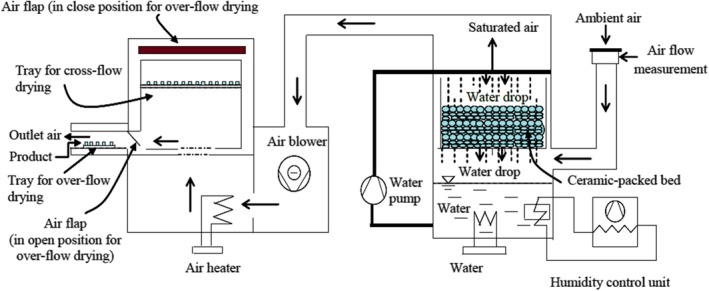
Schematic diagram of the laboratory dryer

The parchment Arabica coffee was cleaned from the mucilage and stored at a temperature of 5°C. The parchment coffee stayed at room temperature for 16 hr before starting the experiment to achieve equilibrium conditions. Thin‐layer drying of the parchment coffees was conducted in a laboratory dryer under controlled temperature and relative humidity conditions. The schematic diagram of the laboratory dryer in Figure 1 was designed and developed by Guarte (1996). This laboratory dryer contains two main sections: a humidifier section and a drying section. The humidifier section consists of a ceramic‐packed bed, a water heater, a water pump, and a humidity control unit. The drying section contains a drying chamber, an air heater, and an air blower. The blower forces air to pass through a humid, ceramic‐packed bed, a porous media, which absorbs and transfers moisture to the air. Then, the air becomes saturated at the same temperature as the heater controls the water. Then, the saturated air temperature is controlled to reduce the relative humidity by the air heater, and the air passes parallel to the parchment coffee. The relative humidity and temperature of the drying process are manually controlled by a psychometric chart with adjustable power supplied by the air heater and water heater.

The dryer was operated before starting an experiment for 1 hr to achieve steady‐state drying conditions. Every experiment was fixed at an air velocity of 1 m/s. The parchment coffee (approximately 100 g) was placed on a tray in a thin layer inside the laboratory dryer. Thin‐layer drying of the parchment coffee was conducted at a temperature and relative humidity in the drying range of 50–70°C and 10%–30%, respectively. Drying air temperature was measured every 5 min with a thermocouple (K type) and was recorded with a data logger. The mass of the parchment coffee was recorded by an electronic balance (accuracy ± .01 g) at an interval of 1 hr. The electronic balance was placed near the drying chamber in the laboratory. The drying process continued until the change in the sample mass was the lowest. All experimental drying conditions were conducted in three replicates.

### 2.2 Mathematical modeling

Models of thin‐layer drying that describe the drying characteristics of biological material consist of three equations: a theoretical equation, a semitheoretical equation, and an empirical equation. The theoretical equation provides the best explanation for the heat and mass transfer of the product during drying, but it is difficult to solve this equation; in addition, the results have low accuracy. The semitheoretical equation is a method for solving the theoretical equation quickly and easily by reducing the form of the answer to the theoretical equation. An empirical equation is easier to calculate than the previous two equations; thus, the model is widely used. The coefficients and various constants of the empirical equation of thin‐layer drying were fitted to the experimental drying data to identify a suitable equation for describing and predicting the drying behavior of the product. The moisture content ratio was defined as:

(1) $$\text{MR =}\;\frac{M_{\text{t}}-M_{\text{e}}}{M_{0}-M_{\text{e}}}$$

where MR: the dimensionless moisture content ratio; *M*_t_: the moisture content at any time (% d.b.); *M*_0_: the initial moisture content (% d.b.); and *M*_e_: the equilibrium moisture content (% d.b.).

The moisture content dry basis was defined as:

(2) $$M=\frac{w(t)-d}{d}\times 100\%$$

where *s* the mass of dried product samples at instant *t* and *d* is the mass of the oven‐dried product samples at 105°C (Helrich, 1990).

For all experimental drying, the final moisture content of the parchment coffee achieved equilibrium under constant conditions of temperature and relative humidity. The nine thin‐layer drying models in Table 1 were selected as suitable models for explaining the drying process of parchment coffee.

**Table 1:** Thin‐layer drying models

Model	Name	Reference
MR = exp(‐kt)	Newton	Mujumdar, 1987
MR = exp(‐kt^n^)	Page	Diamante & Munro, 1993
MR = a exp(‐kt)	Henderson and Pabis	Zhang & Litchfield, 1991
MR = a exp(‐kt) + c	Logarithmic	Yagcioglu, Degirmencioglu, & Cagatay, 1999
MR = a exp(‐kt)** +** b exp(‐gt)	Two‐term	Henderson, 1978
MR = a exp(‐kt)** +** b exp(‐gt)** +** *n* exp(‐ct)	Modified Henderson and Pabis	Karathanos, 1999
MR = a exp(‐kt)** +** (1‐a) exp(‐kat)	Two‐term exponential	Doymaz, 2004
MR = a exp(‐kt)** +** (1‐a) exp(‐kbt)	Approximation of diffusion	Akpinar et al., 2003
MR = exp(‐kt^n^) + bt	Modified Midilli	Ghazanfari&, 2007 EmamiTabil Panigrahi; Ghosh, & Venkatachalapathy, 2014

The mathematical models were adjusted by nonlinear least square regression analysis. In choosing the model, specific values were considered: the coefficient of determination (*r* ^2^) and the root mean square error (RMSE). For the best fit, *r* ^2^ must be the highest, and RMSE must be the lowest. These parameters were defined as follows:

(3) $$r^{2}=1-\frac{\sum_{i=1}^{n}\left(\right.MR_{\mathrm{pre},i}-MR_{\mathrm{obs},i}{)}^{2}}{\sum_{i=1}^{n}\left(\right.M_{\mathrm{obs},i}-MR_{\mathrm{pre},i}{)}^{2}}r^{2}=1-\frac{\sum_{i=1}^{n}\left(\right.MR_{\mathrm{pre},i}-MR_{\mathrm{obs},i}{)}^{2}}{\sum_{i=1}^{n}\left(\right.M_{\mathrm{obs},i}-MR_{\mathrm{pre},i}{)}^{2}}r^{2}=1-\frac{\sum_{i=1}^{n}\left(\right.MR_{\mathrm{pre},i}-MR_{\mathrm{obs},i}{)}^{2}}{\sum_{i=1}^{n}\left(\right.M_{\mathrm{obs},i}-MR_{\mathrm{pre},i}{)}^{2}}$$

(4) $$\mathrm{RMSE}={\left[\frac{\sum_{i=1}^{n}\left(\right.MR_{\mathrm{pre},i}-MR_{\mathrm{obs},i}{)}^{2}}{n}\right]}^{0.5}$$

where *M*_obs,i_: the observed moisture ratios; *M*_pre,i:_ the predicted moisture ratios; and *n*: the number of observations.

### 2.3 Diffusivities of parchment coffee

Fick's second law of diffusion in Equation ((5)) (Crank, 1975) describes a phenomenon of liquid diffusion in the drying of food materials (Aidani et al., 2016; Nilnont et al., 2012; Muhidong et al., 2013; Ghosh, & Venkatachalapathy, 2014; Tolessa, Rademaker, Rademaker, & Boeckx, 2016; Siqueire et al., 2017; Deeto et al., 2018). The cylindrical analytical solution of Equation ((5)) was presented in Equation ((6)), which assumes that the diffusivity of the materials is constant with a negligible reduction (Crank, 1975; McMinn & Magee, 1999; Ramachandran, Paliwal, & Cenkowski., 2018).

(5) $$\frac{\partial M}{\partial t}=\nabla \left(D_{\text{eff}}\left(\nabla M\right)\right)$$

(6) $$\text{MR =}\;\frac{M_{\text{t}}-M_{\text{e}}}{M_{\text{o}}-M_{\text{e}}}=\frac{8r^{2}}{l^{2}}\sum_{i=1}^{\infty }\sum_{j=1}^{\infty }\frac{1}{\lambda_{i}^{2}\beta_{j}^{2}}\exp\left(-\left(\lambda_{i}^{2}+\beta_{j}^{2}\right)\frac{D_{\text{eff}}t}{r^{2}}\right)$$

where *D*_eff_: the effective moisture diffusivity (m^2^/s), *r*: the parchment coffee radius, l: the length of parchment coffee (m), *t*: the drying time (*s*), and *λ_i_*: ith root of the Bessel function (2.405, 5.520, 8.654,...) of zero order, i = 1, 2, 3, and.

(7) $$\beta_{j}=\frac{(2j-1)\pi r}{2l},j=1,2,3$$

The drying time was too long (MR <.6), and Equation ((6)) was reduced to Equation ((8)) because *l* and *r* are small and *t* is large (Crank, 1975), substituting *i* = 1, *j* = 1. Thus, *λ_1_* = 2.4048 for Equation ((8)) (Usub et al., 2010):

(8) $$\text{MR =}\;\frac{M_{\text{t}}-M_{\text{e}}}{M_{\text{o}}-M_{\text{e}}}=\frac{32}{\lambda_{1}^{2}\pi^{2}}\exp\left(-\left(\lambda_{1}^{2}+\beta_{1}^{2}\right)\frac{D_{\text{eff}}t}{r^{2}}\right)$$

From Equation ((8)), the effective moisture diffusivity was computed by minimizing the sum of squares of the deviations between the predicted and the experimental moisture content data (Janjai et al., 2011; Nilnont et al., 2012).

## 3 RESULTS AND DISCUSSION

### 3.1 Drying characteristics of the parchment coffee

**Figure 2:**
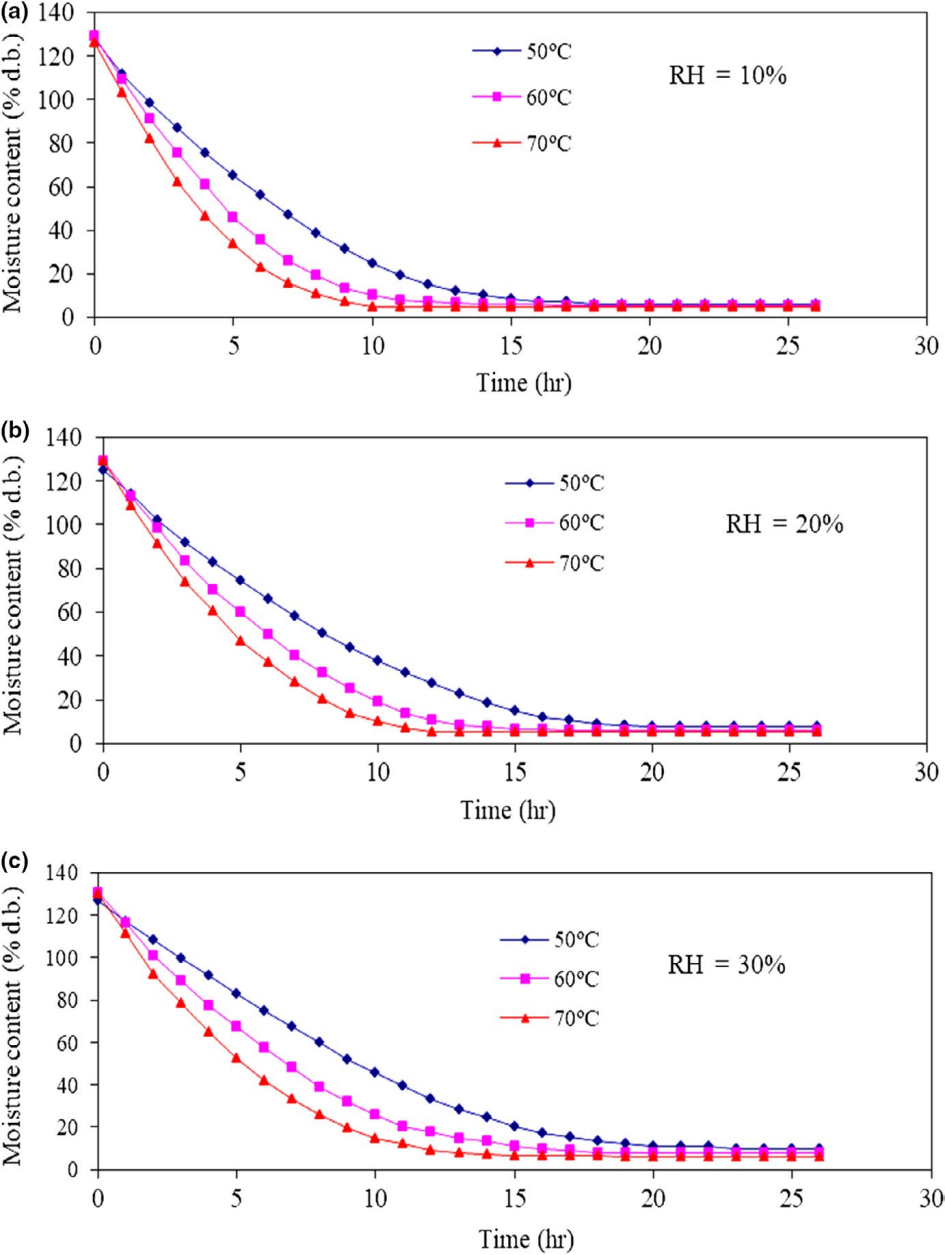
Moisture content of parchment coffee under different drying conditions

All experimental drying conditions were replicated three times, and the average values were used for analysis. The changes in the moisture content of the parchment coffee with time for all drying conditions are shown in Figure 2. The moisture content of the parchment coffee decreased from an initial value of 122% d.b. until the moisture content did not change with time or was constant, which was the final moisture content, ranging from 12.5% d.b. to 4.5% d.b. A decrease in the drying time occurred when the temperature was high because a high drying temperature increases the kinetic energy of a water molecule until it breaks free from the cohesive force. In comparison with low drying temperatures, high drying temperatures can evaporate water in products. From the experiment, drying parchment coffee at an air temperature of 70°C and a relative humidity of 10% was found to obtain the lowest drying time because the vapor pressure of the drying air was changed by controlling the relative humidity of the drying air. The vapor pressure of the drying air was the lowest when the relative humidity was controlled at 10%, and the maximum value of the relative humidity of the drying air was controlled at 30%. The results showed that the vapor pressures are 3.119 kPa, 6.238 kPa, and 9.357 kPa when the relative humidity of the drying air was controlled at 10%, 20%, and 30%, respectively (Cengel & Boles, 2007).

The important factors that affected the reduction in the moisture content on drying were the velocity, temperature, and relative humidity of drying air. This experiment fixed the drying air velocity as constant and controlled the relative humidity at each temperature to study the drying behavior of the parchment coffee. For convective drying, a high air‐drying temperature transfers heat to the product, resulting in a higher temperature, and then, the water within the product moves by diffusion and evaporates into the drying air. Drying at a low relative humidity results in a decrease in the vapor pressure that causes the water inside the product to evaporate rapidly in large quantities. This mass transfer process will stop when the water vapor pressure at the product surface becomes equal to the water vapor pressure of the drying air.

### 3.2 Modeling of parchment coffee

The moisture content based on the data obtained in the experiments was converted to the moisture ratio (MR) and then fitted to the nine thin‐layer drying models in Table 1 to identify a suitable equation for describing and predicting coffee drying behavior during the drying process that controlled the temperature and relative humidity. The estimated parameter values and statistical analysis values (*r* ^2^ and RMSE) are also shown in Table 2. The statistical analysis results showed that of the models, the modified‐Midilli model had the highest *r* ^2^ of .9976 and the lowest RMSE of 6.65%. Therefore, the modified‐Midilli model was considered the best model, followed by the Page model to represent the drying behavior of parchment coffee. For all the drying conditions of these models, the *r* ^2^ value was greater than .9959, indicating a good fit, and the RMSEs were also almost the same. Although the modified‐Midilli model performed the best, this model or the Page model could be considered to describe parchment coffee drying behavior.

**Table 2:** Parameters of the models under different drying conditions

Models	T (ºC)	RH (%)	*k*	*n*	*a*	*b*	*c*	*g*	*r* ^2^	RMSE (%)
Newton	50	10	.13392						.9938	10.89
	60	10	.19754						.9936	12.74
	70	10	.24881						.9926	14.37
	50	20	.11736						.9952	9.8
	60	20	.17297						.9945	11.25
	70	20	.2049						.9946	11.08
	50	30	.09931						.9965	7.24
	60	30	.13797						.9975	6.56
	70	30	.16585						.9966	8.06
Page	50	10	.08745	1.19507					.9982	5.92
	60	10	.16853	1.09016					.9966	8.93
	70	10	.18642	1.18910					.9959	9.14
	50	20	.08138	1.15955					.9988	4.83
	60	20	.13766	1.11952					.9960	9.04
	70	20	.17358	1.09551					.9974	7.22
	50	30	.07314	1.13665					.9994	3.09
	60	30	.11676	1.07778					.9985	5.15
	70	30	.13748	1.09557					.9980	6.54
Henderson and Pabis	50	10	.14055		1.05148				.9952	9.53
	60	10	.20390		1.03238				.9941	11.2
	70	10	.25849		1.04184				.9934	13.44
	50	20	.12295		1.04794				.9965	8.76
	60	20	.17963		1.03927				.9950	10.04
	70	20	.21106		1.03054				.9949	10.59
	50	30	.10318		1.03717				.9975	6.93
	60	30	.14214		1.03012				.9980	6.27
	70	30	.17171		1.03583				.9927	14.73
Logarithmic	50	10	.12863		1.07067		−0.03291		.9960	8.82
	60	10	.22254		1.20207		0.02647		.9957	9.29
	70	10	.27565		1.03181		0.01972		.9946	12.77
	50	20	.11491		1.06325		−0.02564		.9964	7.86
	60	20	.19041		1.03097		0.01825		.9958	9.02
	70	20	.22786		1.02026		0.02311		.9961	8.05
	50	30	.08613		1.09422		−0.08007		.9990	5.32
	60	30	.14607		1.02547		0.00904		.9980	6.93
	70	30	.17958		1.02926		0.01421		.9975	7.11
Two‐term	50	10	.14055		0.52409	0.52738		.14056	.9959	9.72
	60	10	.21174		1.04091	0.00182		−.13073	.9953	10.51
	70	10	.30660		1.30486	−0.30938		.93822	.9950	10.69
	50	20	.12296		0.49996	0.54798		.12293	.9965	8.12
	60	20	.16853		0.51974	0.51952		.17842	.9951	11.03
	70	20	−.14538		0.00115	1.03761		.21732	.9954	10.15
	50	30	.10319		0.51866	0.51861		.10316	.9975	6.90
	60	30	.14215		0.21863	0.81148		.14215	.9981	6.21
	70	30	.17168		0.52233	0.51349		.17184	.9977	7.03
	50	10	.14055		0.52409	0.52738		.14056	.9959	9.72
	60	10	.21174		1.04091	0.00182		−.13073	.9953	10.51
	70	10	.30660		1.30486	−0.30938		.93822	.9950	10.69
	50	20	.12296		0.49996	0.54798		.12293	.9965	8.12
	60	20	.16853		0.51974	0.51952		.17842	.9951	11.03
	70	20	−.14538		0.00115	1.03761		.21732	.9954	10.15
	50	30	.10319		0.51866	0.51861		.10316	.9975	6.90
	60	30	.14215		0.21863	0.81148		.14215	.9981	6.21
	70	30	.17168		0.52233	0.51349		.17184	.9977	7.03
Modified Henderson and Pabis	50	10	.14055	0.13057	0.35049	0.45048	0.34038	.12044	.9956	9.63
	60	10	−.13075	0.51942	0.00182	0.51942	0.52148	.21173	.9963	8.31
	70	10	.25877	0.25878	0.30819	0.31178	0.42187	.25883	.9934	12.44
	50	20	.09203	0.07767	1.34420	1.32691	−1.63801	.09182	.9972	7.47
	60	20	.17966	0.17950	0.28565	0.28663	0.46698	.17972	.9952	9.89
	70	20	.21730	−0.14230	0.51881	0.50770	0.00116	.21736	.9973	7.85
	50	30	.10318	0.10317	0.34519	0.34561	0.34574	.10216	.9975	7.06
	60	30	.14215	0.13214	0.33798	0.33072	0.36140	.15326	.9980	6.04
	70	30	.17152	0.18170	0.31960	0.28950	0.42672	.19181	.9971	7.89
Two‐term exponential	50	10	.18092		1.73886				.998	6.13
	60	10	.21278		1.75026				0.9962	9.84
	70	10	.34289		1.76370				0.9953	10.11
	50	20	.15580		1.69979				.9981	5.86
	60	20	.22502		1.66256				.9959	9.44
	70	20	.26025		1.61890				.9975	8.59
	50	30	.12875		1.64892				.9993	3.35
	60	30	.16814		1.54767				.9983	5.97
	70	30	.20869		1.60495				.9979	8.87
Approximation of diffusion	50	10	.23916		−4.29830	0.87914			.9981	5.89
	60	10	.20205		0.99909	−0.78070			.9955	11.43
	70	10	.25284		0.99097	−0.59121			.9944	12.12
	50	20	.33056		−0.3863	0.44253			.9985	5.78
	60	20	.15396		3.60075	0.95726			.9945	11.94
	70	20	.20866		0.99936	−0.79476			.9966	10.53
	50	30	.09903		0.49315	1.00044			.9965	10.97
	60	30	.14997		1.09583	1.07955			.9985	5.6
	70	30	.14350		1.96031	0.98243			.9967	8.98
Modified Midilli	50	10	.082430	1.249165		0.001647			.9989	4.3842
	60	10	.139781	1.230308		0.002175			.9989	5.5829
	70	10	.165239	1.297477		0.001333			.9993	5.6216
	50	20	.076210	1.225808		0.001566			.9991	3.7486
	60	20	.121461	1.179043		0.001722			.9997	2.323
	70	20	.149130	1.213382		0.001422			.9996	3.6049
	50	30	.055194	1.300503		0.002104			.9992	3.0454
	60	30	.098322	1.238872		0.002484			.9992	4.049
	70	30	.138045	1.206457		0.001858			.9994	3.9583

**Figure 3:**
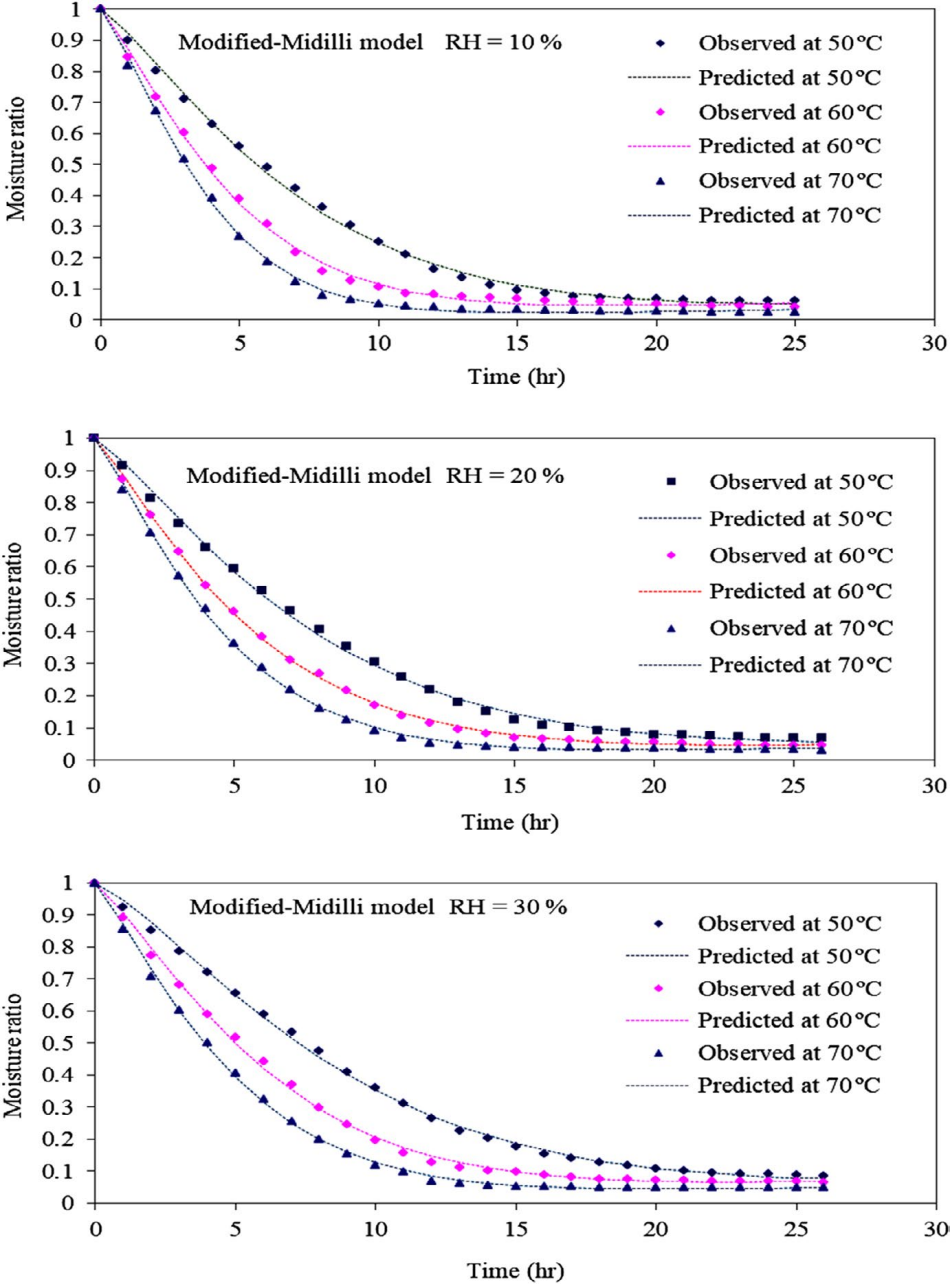
Variation in parchment coffee moisture ratio using the modified‐Midilli model under different drying conditions

**Figure 4:**
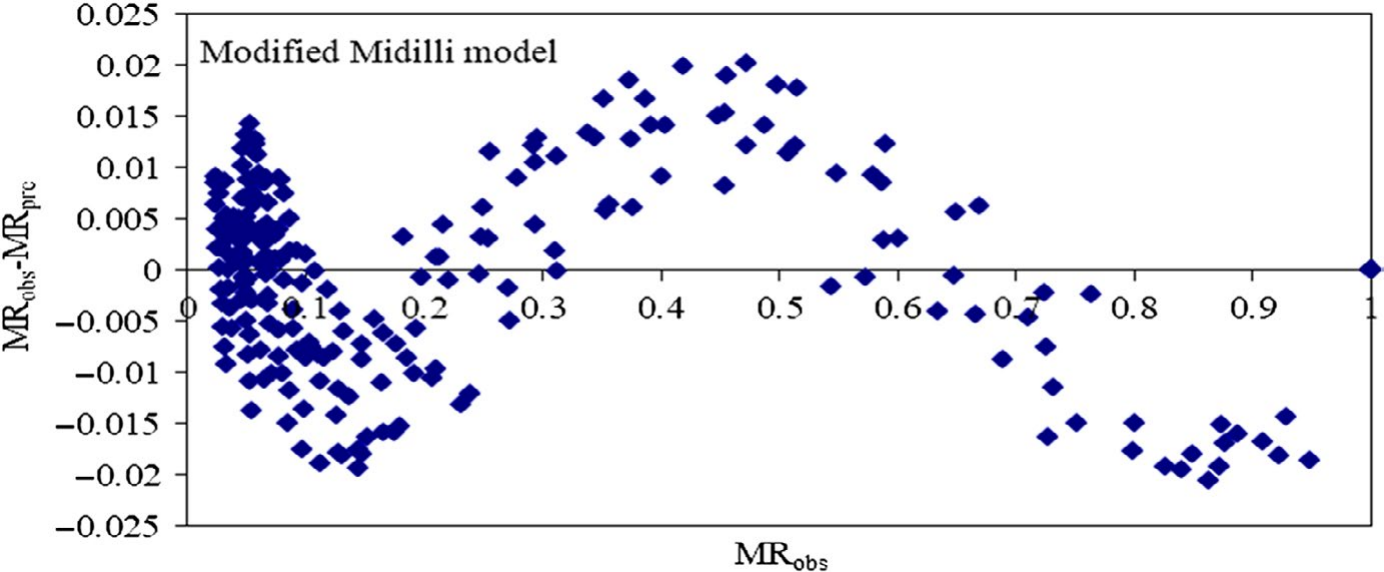
Residual plot of observed moisture content (Mobs) and predicted moisture content (Mpre) of parchment coffee from the modified‐Midilli model under different drying conditions

The correlations between the experimental and predicted data of the modified‐Midilli model for parchment coffee drying are shown in Figure 3, which presents the variation in the parchment coffee MRs with drying time under different drying conditions. The MR decreases with time, and the difference among the MRs increased continuously from the beginning to the end of drying. The observed and predicted values of the model are perfectly consistent and almost the same. Residual plots, which were the differences between the observed moisture content and the predicted moisture content, were used to consider the selected model and are shown in Figure 4. The randomness of the residual plots showed that there was no systematic pattern, demonstrating the suitability of the derived models.

**Figure 5:**
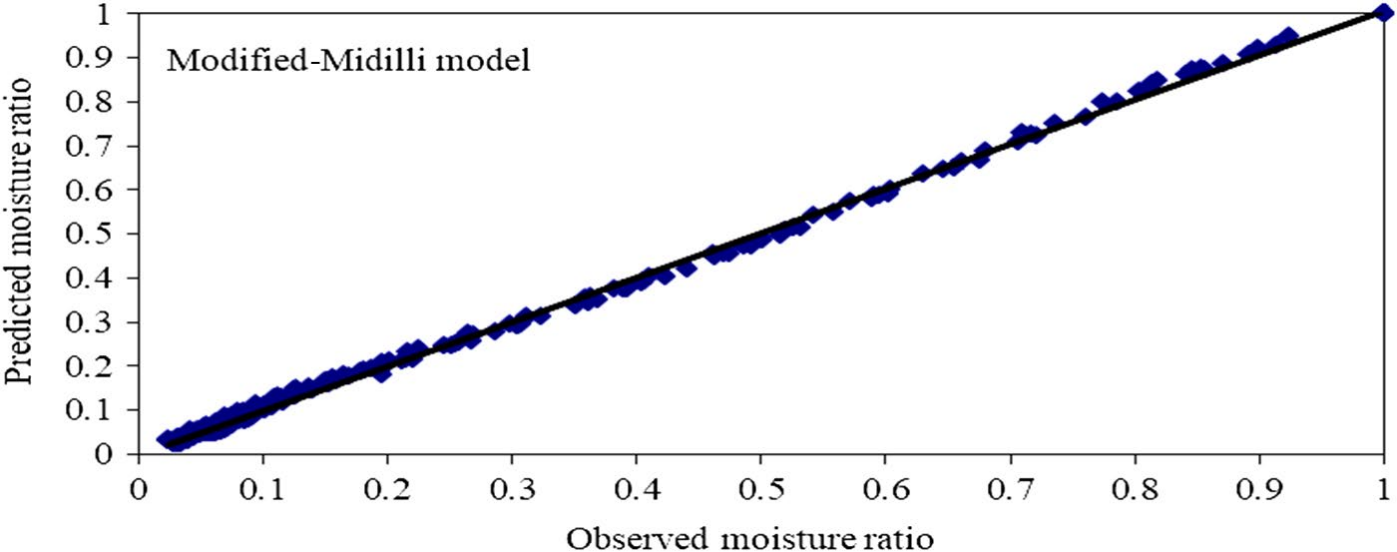
The correlations of the observed and the predicted data of the modified‐Midilli model under different drying conditions

The plot of the observed data and the predicted data of the modified‐Midilli model for different conditions is shown in Figure 5. All data are banded around the straight lines. This result indicates that modified‐Midilli models are powerful in predicting the drying behavior of parchment coffee within the drying air temperature range of 50–70°C. This model was simplified because it expresses the MR of parchment coffee as a function of empirical parameters.

The drying constant values of the modified‐Midilli model for all drying conditions are presented in Table 2. The value of the drying constant “*k*” increased with increasing drying temperature. The constant values “*k*,” “*n*,” and “*b*” of the modified‐Midilli model were regressed by nonlinear least square regression analysis with respect to the temperature and relative humidity of the drying air. These values can be calculated through the following expression in the form of a second‐order polynomial, as shown in Table 3.

**Table 3:** Equations of drying of parchment coffee

Model	Equations of drying parameters	*r* ^2^
Modified Midilli	*k* = −.41202 + .014550T − 6.3162 × 10^−4^RH + 1.0318 × 10^−4^TRH – 8.8133 × 10^−5^T^2^ – 2.4318 × 10^−5^RH^2^	.9955
	*n* = 2.19467 − .033121T + .001747RH − .000356TRH + .000327T^2^ + .000477RH^2^	.9856
	*b* = −.01318 + 5.5127 × 10^−4^T − 1.3408 × 10^−4^RH + 1.7094 × 10^−7^TRH − 4.7207 × 10^−6^T^2^ + 3.6350 × 10^−6^RH^2^	.9660

### 3.3 Effective moisture diffusivity

According to the drying experiment results, the mechanism of diffusion could be analyzed through Fick's second law for cylindrical shapes. The effective moisture diffusivities of parchment coffee for all conditions are presented in Table 4, with values ranging between 7.7554 × 10^–10^ and 1.4525 × 10^–9^ m^2^/s. These values are within the range of diffusivities (10^−11^ m^2^/s–10^−9^ m^2^/s) for different crops using several drying methods (McMinn & Magee, 1999), such as the moisture diffusivity of coffee ranging from 7.17 × 10^−10^ to 10.00 × 10^−10^ m^2^/s for drying air temperatures ranging from 40 to 60°C (Nilnont et al., 2012; Varadharaju, Karunanidhi, & Kailappan, 2001). For convective drying, a high drying air temperature transfers heat to the product, resulting in a higher product temperature, and then, the water within the product moves by diffusion and evaporates into the drying air. Drying at a low relative humidity decreases the vapor pressure causing the water inside the product to evaporate rapidly in large quantities. This mass transfer process will stop when the water vapor pressure at the product surface becomes equal to the water vapor pressure of the drying air. Therefore, the drying air temperature and relative humidity have an essential effect on the drying of parchment coffee: the effective moisture diffusivity of parchment coffee increased with a decrease in the relative humidity and an increase in the temperature of drying air.

**Table 4:** The effective moisture diffusivity of parchment coffee

Temperature (°C)	The effective diffusivity (m^2^/s)			Mean effective diffusivity (m^2^/s)
	RH = 10%	RH = 20%	RH = 30%	
50	9.8074 × 10^–10^	8.6104 × 10^–10^	7.7554 × 10^–10^	8.7240 × 10^–10^
60	1.2231 × 10^–9^	8.9110 × 10^–10^	8.2473 × 10^–10^	9.7963 × 10^–10^
70	1.4525 × 10^–9^	9.4980 × 10^–10^	8.5564 × 10^–10^	1.0859 × 10^–9^

**Figure 6:**
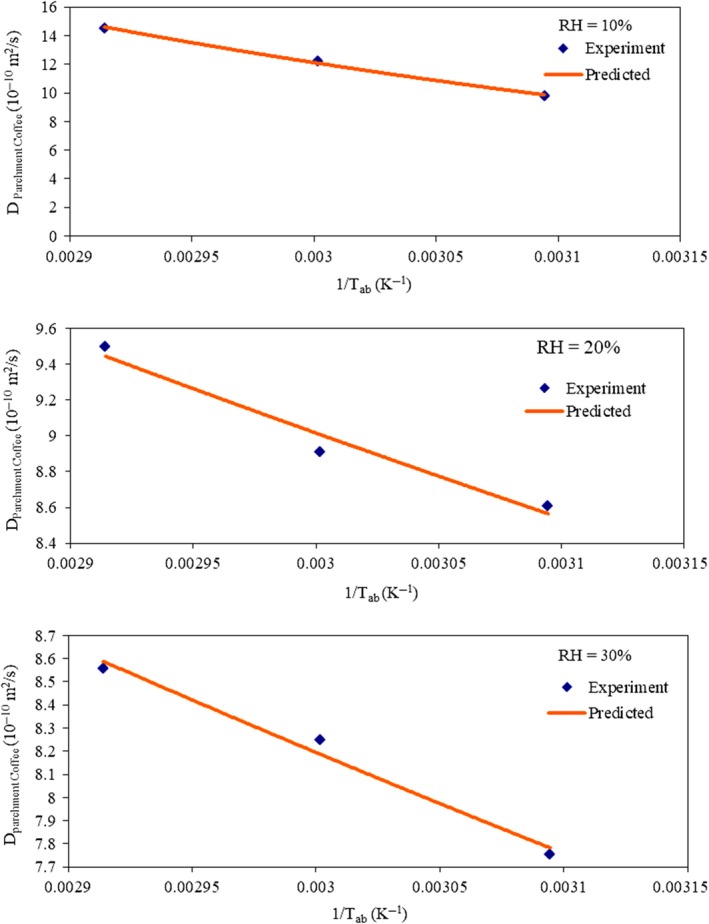
Effective moisture diffusivity of parchment coffee as a function of the reciprocal of absolute drying air temperature (Tab) at different relative humidities (10%–30%)

Figure 6 shows the variations in moisture diffusivities of parchment coffee as functions of the reciprocal of absolute drying air temperature. The effective diffusivities of parchment coffee for drying air temperatures of 50–70°C and various relative humidities (10%–30%) were found to be dependent on temperature and can be determined as a function of temperature using the Arrhenius‐type equations as given in Equations (9), (10), (11):

For RH = 10%

(9) $$D_{\text{parchment}\;\text{coffee}}=8.0\times 10^{-7}\;{\text{e}}^{\left(-2179.9/T_{\text{ab}}\right)},\;r^{2}=.997$$

For RH = 20%

(10) $$D_{\text{parchment}\;\text{coffee}}=5.0\times 10^{-9}\;{\text{e}}^{\left(-542.28/T_{\text{ab}}\right)},\;r^{2}=.9647$$

For RH = 30%

(11) $$D_{\text{parchment}\;\text{coffee}}=4.0\times 10^{-9}\;{\text{e}}^{\left(-546.25/T_{\text{ab}}\right)},\;r^{2}=.9841$$

where *T*_ab_ is the absolute drying air temperature.

## 4 CONCLUSIONS

The results of the coffee drying experiment under different conditions showed that relative humidity affected reductions in moisture content and decreased at each of the drying air temperatures. The temperature of saturated water within the product was dependent on the vapor pressure of the drying air because vapor pressure affects the boiling point of water, which results in the water inside a product evaporating rapidly in large quantities. Thus, the mass transfer caused by the evaporation of water in the product to the drying air was greater under a relative humidity of 10% and a temperature of 70°C than under other conditions.

The maximum effective moisture diffusivity of a drying temperature of 70°C and relative humidity of 10% was 1.4525 × 10^–9^ m^2^/s, which was determined by minimizing the sum of squares. Nine thin‐layer drying models were used to describe the process during parchment coffee drying. Drying parameters were found to be a function of drying air temperature and relative humidity. The agreement between the predicted and experimental data for parchment coffee in the modified‐Midilli model was excellent for considering the drying behavior of parchment coffee, and this model was used optimize the dryer.

## INFORMED CONSENT

None.

## CONFLICT OF INTEREST

None declared.

## ETHICAL STATEMENT

This study does not involve any human nor animal testing.
